# Reconsidering Anesthesia in Lumbar Surgery: An Umbrella Review of Awake Versus General Anesthesia

**DOI:** 10.3390/jcm14238335

**Published:** 2025-11-24

**Authors:** Favour C. Ononogbu-Uche, Carl Tchoumi, Nolan M. Stubbs, Arnav Sharma, Raymond J. Gardocki, Alok Sharan, Muhammad M. Abd-El-Barr, Ernest E. Braxton, Awake Spine Research Group

**Affiliations:** 1 Department of Neurosurgery, Duke University, Durham, NC 27710, USA; favour.ononogbu-uche@downstate.edu (F.C.O.-U.); arnav.sharma@duke.edu (A.S.); muhammad.abd.el.barr@duke.edu (M.M.A.-E.-B.); 2 College of Osteopathic Medicine, Rocky Vista University, Ivins, UT 84738, USA; carl.tchoumi@ut.rvu.edu; 3 Department of Neurosurgery, Rutgers New Jersey Medical School, Newark, NJ 07103, USA; nms324@njms.rutgers.edu; 4 Department of Orthopaedics, Vanderbilt University Medical Center, Nashville, TN 37232, USA; raymond.gardocki@vumc.org; 5 Spine and Performance Institute, Edison, NJ 08820, USA; aloksharan75@gmail.com; 6 Vail Summit Orthopaedics and Neurosurgery, Vail, CO 81657, USA

**Keywords:** enhanced recovery after surgery, general anesthesia, lumbar spine surgery, spinal anesthesia, postoperative outcomes

## Abstract

**Background/Objectives**: Lumbar degenerative disease drives numerous elective spine surgeries, and anesthetic choice significantly influences airway risk, hemodynamics, analgesia, mobilization, and recovery. Interest in awake lumbar surgery, typically using spinal anesthesia (SA) with light sedation, has grown as comparative studies suggest comparable safety to general anesthesia (GA) with potential reductions in opioid use, nausea, time to ambulation, and efficiency metrics. However, these benefits may be context-dependent under standardized perioperative care. Therefore, the aim of this umbrella review is to synthesize previously published meta-analyses that compare postoperative outcomes between SA and GA in patients undergoing lumbar spine surgery. **Methods**: A systematic literature search was executed with defined criteria across PubMed, Embase, and Web of Science. Data analysis was performed using the metaumbrella R package to report equivalent Hedges’ g values. Each meta-analysis was evaluated with the AMSTAR2 tool, and the credibility of the evidence was determined with Ioannidis criteria. **Results**: Seven meta-analyses were included. Pooled data showed that SA was associated with shorter operative time, reduced length of stay, and lower intraoperative blood loss, supported by class III credibility for operative time and length of stay and class IV for blood loss in the setting of high between study heterogeneity. SA was also associated with lower odds of postoperative nausea and vomiting and reduced postoperative analgesic requirements, both graded as class IV with prediction intervals that encompassed the null. Intraoperative hypotension and bradycardia did not differ significantly between SA and GA, and postoperative pain scores and overall complication rates were similarly neutral. **Conclusions**: This umbrella review identifies potential advantages of SA in lumbar spine surgery, including shorter operative time, reduced length of stay, lower intraoperative blood loss, and lower postoperative nausea and analgesic requirements, while finding no consistent differences in hemodynamic events or overall complications. These findings suggest SA as an alternative pathway to general anesthesia for selected lumbar procedures but highlight substantial heterogeneity and low-to-intermediate credibility for several endpoints, underscoring the need for additional high-quality, protocolized comparative studies to refine effect sizes and define optimal patient and procedural selection.

## 1. Introduction

Lumbar degenerative pathology is a leading driver of elective spine surgery, and anesthetic modality is a modifiable determinant of airway risk, hemodynamics, analgesia, mobilization, and recovery [1]. Interest in “awake” lumbar surgery, most often spinal anesthesia with light sedation, has increased for decompressions and select fusions provided to patients within fast-track care pathways [2]. As outpatient spine surgery expands and hospitals face pressure to reduce lengths of stay, rapid anesthetic recovery has become a major priority [3]. Multiple syntheses and comparative studies suggest that, in appropriately selected patients, neuraxial techniques can match the safety of general anesthesia while lowering opioid use, postoperative nausea and vomiting, and time to ambulation, with potential reductions in operative or anesthesia time and length of stay in some settings [1,2,3,4]. Small randomized and cohort studies broadly support these advantages, although effect sizes differ by procedure and institutional pathway [5,6]. In contrast, large database analyses indicate that benefits may be modest or context-dependent after risk adjustment, implying that patient selection, surgical technique, and perioperative protocols mediate observed effects [7]. Enhanced Recovery After Surgery (ERAS) programs for lumbar decompressions and fusion elevate the relevance of anesthetic choice because they standardize multimodal, opioid-sparing analgesia and early mobilization, yet they do not mandate a single anesthetic across indications, leaving clinicians to interpret heterogeneous evidence when designing pathways and counseling patients [8]. Together, these observations raise practical questions for surgeons and anesthesiologists about when and for whom awake techniques should be preferred over general anesthesia to maximize recovery and value.

The evidence base includes multiple overlapping systematic reviews and meta-analyses that differ in eligibility criteria, outcome definitions, and analytic choices across microdiscectomy, endoscopic and minimally invasive decompression, and emerging reports of awake fusion. These discrepancies limit clear guidance as ERAS elements, outpatient pathways, and procedure mix continue to evolve. An umbrella review is well suited for addressing this gap because it collates and appraises review-level evidence, maps overlap in primary studies, and grades credibility per outcome, allowing comparison across syntheses and identification of where further trials or better standardization are needed [9,10]. Our aim is to conduct a systematic umbrella review of published systematic reviews and meta-analyses comparing awake, primarily with neuraxial anesthesia, lumbar surgery with lumbar surgery under general anesthesia, to quantify the consistency and strength of evidence for perioperative efficiency, complications, and patient-centered recovery and to test how the procedure class and ERAS context modify effects. By elevating the unit of analysis to the review level and applying the established umbrella methodology, this work seeks to provide an evidence-graded summary to inform case selection, pathway design, and research priorities for lumbar spine surgery [8,9].

## 2. Materials and Methods

**Figure 1:**
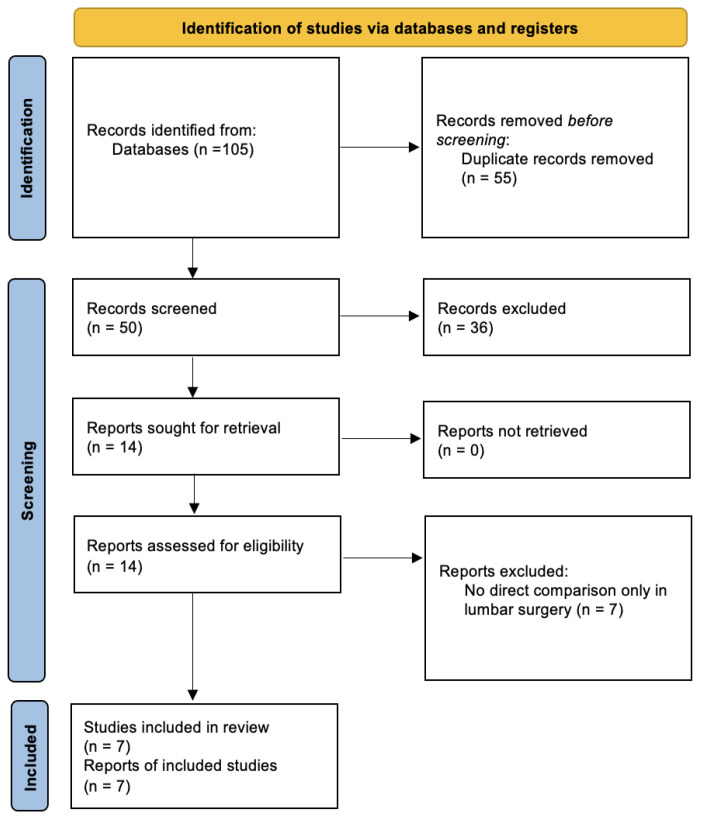
PRISMA flow diagram.

This umbrella review and its design adhered to the PRIOR checklist [9] and Preferred Reporting Items for Systematic Review and Meta-Analyses (PRISMA) guidelines (Figure 1). A prior protocol was not prepared or registered. Eligibility was defined through the PICOS framework. The population (P) consisted of patients of any age undergoing lumbar spine procedures. The intervention (I) was awake anesthesia, defined as spinal, epidural, or local anesthesia with or without light sedation, and the comparator (C) was surgery performed under general anesthesia. The outcomes of interest (O) were operative time, intraoperative blood loss, length of stay (LOS), postoperative pain assessed through the visual analog scale (VAS), surgical complications, postoperative nausea and vomiting (PONV), analgesic requirements, intraoperative hypotension, and intraoperative bradycardia. The study design (S) was restricted to published meta-analyses. Studies were not considered if they (1) were duplicates; (2) had an absence of quantitative meta-analytic data, such as qualitative data syntheses (case reports, reviews, or observational studies); (3) were poorly supported by primary data; (4) did not report any of the predefined outcomes of interest; (5) were not published in the English language. The completed PRISMA checklist is included in the Supplementary Materials (Table S1).

### 2.1. Study Selection

After an extensive literature search was performed across PubMed, Embase, and Web of Science, all identified citations were screened to remove duplicates. Search terms included terms for (1) lumbar surgery: ((lumbar OR “lumbar spine” OR lumbosacral) AND (decompression OR laminectomy OR discectomy OR microdiscectomy OR endoscopic OR microendoscopic) and (2) anesthesia modality: (“spinal anesthesia” OR “spinal anesthesia” OR neuraxial OR “regional anesthesia” OR “local anesthesia” OR awake OR “conscious sedation”) AND (“general anesthesia” OR GA)).

Two independent reviewers screened titles and abstracts to determine eligibility based on the inclusion criteria. Full texts of the articles that met these criteria were retrieved. Both reviewers analyzed the full texts of the studies to determine fit with the inclusion criteria. Cited-reference searching was performed. A third reviewer mediated conflicts between the two primary reviewers throughout screening.

### 2.2. Data Extraction

Extraction was performed by two independent reviewers. Disagreements in data extraction or risk of bias ratings were settled by consulting a third reviewer. From each included meta-analysis, these data were collected: first author and publication year, date of the last literature search, databases or data sources used, publication range of the primary studies, total number of included studies, total pooled sample size, numbers of patients in the spinal and general anesthesia groups, the effect measure applied, pooled effect estimates with 95% confidence intervals (*CIs*), *p* values for the pooled effects, *I*^2^ statistics and corresponding *p* values for heterogeneity, and any reported assessments of publication bias. When available in the meta-analyses, we also recorded primary study level characteristics as reported, including publication year and study design, total sample size, group-specific sample sizes for awake and asleep anesthesia, and individual study effect estimates with 95% CIs.

### 2.3. Quality Assessment

The methodological quality of each included meta-analysis was assessed using the AMSTAR 2 (A Measurement Tool to Assess Systematic Reviews) instrument, and any differences in ratings were resolved via discussion among the authors.

### 2.4. Data Analysis and Credibility of the Evidence for the Outcomes of Interest

This project was conducted as an umbrella review, with published systematic reviews and meta-analyses as the unit of analysis; primary trials were not re-pooled, and review-level effect estimates were synthesized using the metaumbrella R package (Version 4.5). Characteristics, findings, and quality ratings of the included meta-analyses were summarized using descriptive methods. For continuous outcomes, we extracted or derived standardized mean differences as Hedges g with 95 percent confidence intervals, and for dichotomous outcomes, we used odds ratios with 95 percent confidence intervals, harmonizing directions so that benefits with awake corresponded to negative g for undesirable metrics, such as length of stay, operative time, blood loss, and pain, and to odds ratios below 1 for adverse events such as hypotension, bradycardia, PONV, and complications. All meta-analyses used random effects models with inverse variance weighting, and we reported pooled effects together with between-study heterogeneity as *I*^2^ and 95 percent prediction intervals to characterize expected effects in new settings. Small-study and reporting biases were evaluated using Egger’s regression test and the excess significance bias test, with robustness assessed through leave-one-out jackknife analyses and largest study influence checks. The credibility of evidence for each outcome was graded using Ioannidis classes, and the power to detect a medium effect size was recorded. Data quality checks flagged non-symmetric confidence intervals, which we resolved by verifying that the effect size was the midpoint on the appropriate scale and by computing odds ratio limits on the log scale before back transformation to ensure numeric consistency. For dichotomous outcomes, we expressed effects as odds ratios and harmonized all estimates so that values less than 1.0 indicated lower odds of the event with awake anesthesia and values greater than 1.0 indicated lower odds with general anesthesia. When original meta-analyses reported odds ratios for the absence of an event, we inverted these estimates to align with this convention. Planned sensitivity analyses included rerunning models after correction of problematic rows, exploring subgroups by procedure and anesthetic adjuncts, and confirming that conclusions were unchanged when direction conventions were varied. Meta-analyses that produced materially non-symmetric or otherwise implausible confidence intervals around the pooled effect and could not be corrected on the appropriate scale were excluded from the umbrella synthesis.

## 3. Results

### 3.1. Literature Search

The literature search yielded 105 records. After 55 duplicates were removed, 36 were excluded after screening titles and abstracts. Seven articles were excluded after the full-text screening. We selected seven meta-analyses to be included in our study (Figure 1).

### 3.2. Meta-Analysis Characteristics

All seven included meta-analyses compared postoperative outcomes between awake lumbar surgery and general anesthesia counterpart in patients of any age [1,2,11,12,13,14,15]. Meta-analyses included 8 to 38 primary studies, were published between 2017 and 2023, and had total sample sizes of 625 to 3709. Characteristics across the seven included studies are listed in Table 1.

### 3.3. Quality Assessment

Each of the seven meta-analyses demonstrated high methodological quality (mean AMSTAR 2 score: 9.86). Individual AMSTAR 2 assessments per included meta-analysis are reported in Table 2.

### 3.4. Summary of Outcomes

**Figure 2:**
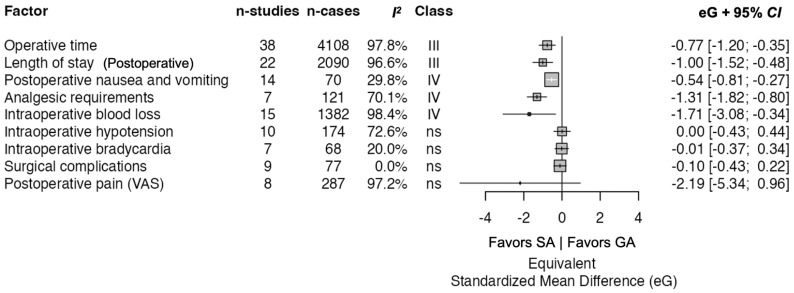
Forest plot of the pooled outcomes. SA indicates spinal anesthesia; GA indicates general anesthesia.

There outcomes of interest were extracted from the included studies: operative time, intraoperative blood loss, LOS, postoperative pain, surgical complications, PONV, analgesic requirements, intraoperative hypotension, and intraoperative bradycardia. These outcomes are summarized in Table 3. Pooled analyses results are depicted in Table 4 and visualized in Figure 2.

### 3.5. Operative Time

Four meta-analyses (38 studies, 7029 patients) compared awake versus general anesthesia for operative time. The pooled effect demonstrated decreased operative time in the awake group and was statistically significant [eG = −0.773, 95% CI: −1.197 to −0.349, *p* = 3.57 × 10^−4^]. Heterogeneity was high (*I*^2^ = 97.8%, *p* < 0.001). No small-study effects were detected by Egger’s test (*p* = 0.548), and there was no excess significance bias (*p* = 1.00). Jackknife removal retained significance (*p* = 7.64 × 10^−4^). Credibility was graded as class III.

### 3.6. Intraoperative Blood Loss

Three meta-analyses (15 studies, 2721 patients) evaluated blood loss. Awake anesthesia favored lower blood loss, with a significant pooled effect [eG = −1.708, 95% CI: −3.081 to −0.335, *p* = 0.0147]. Heterogeneity was very high (*I*^2^ = 98.4%, *p* < 0.001), and the prediction interval crossed the null (−7.675 to 4.259). No small-study effects were detected (Egger *p* = 0.251), and no excess significance bias was detected (*p* = 0.342). Jackknife remained significant (*p* = 0.034). Credibility was graded as class IV.

### 3.7. Length of Stay

Four meta-analyses (22 studies, 3965 patients) reported postoperative hospital length of stay during the index admission, generally in days. Awake anesthesia reduced length of stay with a significant pooled effect [eG = −1.003, 95% CI: −1.522 to −0.484, *p* = 1.50 × 10^−4^]. Heterogeneity was high (*I*^2^ = 96.6%, *p* < 0.001). Egger’s test did not indicate small-study effects (*p* = 0.259), but excess significance bias was present (*p* = 6.25 × 10^−8^). Jackknife removal retained significance (*p* = 4.88 × 10^−4^). Credibility was graded as class III.

### 3.8. Postoperative Pain (VAS)

Eight studies, with 672 patients, evaluated postoperative pain using VAS. The pooled effect was not statistically significant [eG = −2.189, 95% CI: −5.338 to 0.96, *p* = 0.173]. Heterogeneity was very high (*I*^2^ = 97.2%, *p* < 0.001), and the prediction interval crossed the null (−13.548 to 9.169). Egger’s test suggested small-study effects (*p* = 0.0019), and excess significance bias testing was non-informative here (*p* = 1.00). Credibility was graded as nonsignificant.

### 3.9. Surgical Complications

Nine studies, with 188 patients, reported overall surgical complications. No significant difference was found [eOR = 0.828, 95% CI: 0.456 to 1.501, *p* = 0.533]. Heterogeneity was absent (*I*^2^ = 0.0%, *p* = 0.62). Egger’s test was negative (*p* = 0.543), and no excess significance bias was detected (*p* = 0.842). Credibility was graded as nonsignificant.

### 3.10. Postoperative Nausea and Vomiting

Two meta-analyses (14 studies, 279 patients) assessed postoperative nausea and vomiting. Awake anesthesia was associated with lower odds of nausea and vomiting [eOR = 0.376, 95% CI: 0.228 to 0.618, *p* = 1.16 × 10^−4^]. Heterogeneity was low to moderate (*I*^2^ = 29.8%, *p* = 0.12), and the prediction interval crossed the null (0.130 to 1.085). No small-study effects were detected (Egger *p* = 0.735), and excess significance bias testing was non-informative. Based on framework thresholds, credibility was graded as class IV.

### 3.11. Postoperative Analgesic Requirements

Three meta-analyses (7 studies, 520 patients) reported analgesic requirements. Awake anesthesia reduced analgesic requirements with a significant pooled effect [eOR = 0.093, 95% CI: 0.037 to 0.234, *p* = 4.58 × 10^−7^]. Heterogeneity was high (*I*^2^ = 70.1%, *p* < 0.001), and the prediction interval crossed the null (0.006 to 1.420). Egger’s test did not indicate small-study effects (*p* = 0.215), and there was no excess significance bias detected (*p* = 0.272). Jackknife removal retained significance (*p* = 6.99 × 10^−5^). Credibility was graded as class IV.

### 3.12. Intraoperative Hypotension

Three meta-analyses (10 studies, 336 patients) reported intraoperative hypotension. The pooled effect did not demonstrate a significant difference between groups [eOR = 1.008, 95% CI: 0.459 to 2.214, *p* = 0.984]. Heterogeneity was high (*I*^2^ = 72.6%, *p* < 0.001), and the prediction interval was wide and crossed the null (0.082 to 12.456). Egger’s test was negative for small-study effects (*p* = 0.973), and no excess significance bias was detected (*p* = 0.307). Jackknife analysis remained nonsignificant (*p* = 0.998). Credibility was graded as nonsignificant.

### 3.13. Intraoperative Bradycardia

Two meta-analyses (7 studies, 150 patients) assessed bradycardia. There was no significant difference between groups [eOR = 0.977, 95% CI: 0.512 to 1.864, *p* = 0.943]. Heterogeneity was low (*I*^2^ = 20.0%, *p* = 0.28). Egger’s test was negative (*p* = 0.800). Credibility was graded as nonsignificant.

## 4. Discussion

Awake anesthesia for lumbar surgery aims to preserve physiological stability, streamline perioperative flow, and reduce resource use by avoiding airway instrumentation and high doses of hypnotics and volatile agents [3,16]. The approach aligns with enhanced recovery principles that emphasize hemodynamic control, multimodal analgesia, and early mobilization, all of which plausibly shorten length of stay and operative time while limiting blood loss [16]. This umbrella review synthesizes a diverse evidence base, suggesting spinal anesthesia’s potential benefits in these areas while clarifying where advantages are uncertain or protocol dependent. Awake anesthesia reduced postoperative length of stay, operative time, and intraoperative blood loss, with class III–IV credibility, although very high heterogeneity and wide prediction intervals indicate that the magnitude of these gains varies across institutions. Postoperative analgesic requirements and postoperative nausea and vomiting were both lower with awake techniques, with statistically robust pooled effects but class IV credibility and prediction intervals that crossed the null, suggesting that local multimodal analgesia and antiemetic protocols likely shape the benefit achieved. Intraoperative hypotension no longer showed a consistent difference between groups after harmonizing effect directions, and high heterogeneity with a broad prediction interval suggests that hemodynamic responses depend more on local dosing strategies and co-administered agents than on the awake state itself.

Across several continuous outcomes, particularly operative time, length of stay, intraoperative blood loss, and early pain scores, pooled effects favored awake techniques but were accompanied by very high between-study heterogeneity (*I*^2^ around 95 to 98 percent) and wide prediction intervals. This pattern suggests that the average direction of effect is consistent, but the magnitude of benefit varies substantially across settings. For example, the standardized mean difference (SMD) for operative time (G −0.77) would correspond roughly to a 15 to 30 minute reduction in operative time in cohorts where baseline procedures last about 120 min with a standard deviation of 20 to 40 min, and the SMD for length of stay (G −1.00) is compatible with a reduction of approximately 0.5 to 1 hospital day when the standard deviation is in that range. At the same time, the wide prediction intervals indicate that some future institutions may see only small gains or minimal differences, particularly if existing asleep pathways are already highly optimized. Clinically, we interpret these findings as supporting a probable efficiency and recovery advantage of awake pathways on average, while emphasizing that local implementation, case mix, and perioperative protocols are likely to determine the exact magnitude of benefit in individual centers.

### 4.1. Clinical Contexts

Selection of an anesthetic pathway for lumbar spine surgery should balance intraoperative physiological stability, efficient and predictable workflow, and a safe, swift recovery. Awake anesthesia advances these goals by avoiding airway instrumentation, deep sedation, and large hemodynamic swings, aligning with enhanced recovery principles that emphasize multimodal analgesia and early mobilization [17]. In this umbrella review, awake anesthesia was prespecified as the intervention and general anesthesia as the comparator. Effect directions were harmonized so that lower length of stay, shorter operative time, less blood loss, and lower odds of postoperative nausea and vomiting or rescue analgesic use indicate benefits with awake techniques. Random-effects models were applied, with heterogeneity metrics and prediction intervals used to estimate expected performance in new settings. The objective is to identify advantages that are likely reproducible in routine practice, highlight protocol-sensitive outcomes, and guide implementation elements that support consistent patient benefit. Importantly, these signals should be regarded as hypothesis-generating and contingent on confirmation in future prospective studies and additional review-level syntheses.

### 4.2. Observed Benefits of Awake Lumbar Surgery

Four findings are directly relevant to clinical decision making. First, length of stay was shorter with awake pathways by about one standard deviation on average. Although standardized mean differences do not map one-to-one to hours, a shift of this magnitude typically corresponds to visibly earlier discharge for common lumbar procedures when pathways are mature and discharge criteria are standardized [14]. Second, operative time was shorter by roughly three quarters of a standard deviation, a pattern that often reflects incremental operational gains, such as simpler room setup, faster transitions between anesthetic milestones, and fewer delays during recovery room clearance [1]. Third, intraoperative blood loss was lower by approximately one and two thirds standard deviation on average. Mechanistically, lighter sedation and fewer hypertensive spikes can reduce venous congestion and facilitate hemostasis, while experienced teams may achieve quicker exposure and closure that further limits bleeding [18]. Fourth, awake techniques were associated with lower postoperative nausea and vomiting and markedly reduced postoperative analgesic requirements, with statistically robust pooled effects, albeit with class IV credibility and prediction intervals that encompassed the null. These symptom and resource use benefits are suggestive of reduced exposure to volatile agents and systemic opioids and suggest that well-executed awake pathways can lessen postoperative discomfort and opioid needs for many patients [19]. Considered together, these results indicate that awake pathways may improve both clinical stability and efficiency when implemented in a protocolized fashion.

### 4.3. Clinical Implications and Future Research

Our results suggest that awake neuraxial techniques may provide net clinical benefits in routine lumbar decompressions and short, single-level fusions in otherwise stable patients, particularly in ambulatory or short-stay settings where a shorter length of stay, reduced operative time, lower blood loss, and less postoperative nausea and analgesic use translate directly into throughput and recovery gains. These observations are consistent with contemporary case series and institutional experiences that have implemented standardized awake lumbar pathways and reported favorable hemodynamics, faster room turnover, and high patient satisfaction in appropriately selected cases [20]. Future prospective studies should therefore prioritize these indications, with clear inclusion criteria that focus on primary degenerative pathology, limited fusion extents, and patients in whom avoiding intubation, deep volatile anesthesia, and high opioid exposure is likely to change risk–benefit balance.

At the same time, several anatomic and anesthetic constraints show the need for careful case selection and explicit safety planning. Prone positioning inherently limits airway access, so awake programs must maintain a low threshold and preplanned strategy for emergent intubation that includes head repositioning, table adjustments, and rapid conversion to general anesthesia when needed. Dense motor blockade from spinal anesthesia precludes the use of motor evoked potentials, which remains standard in many deformity and complex fusion practices, and any sympathectomy-related hypotension may interact with prone positioning to increase the theoretical risk of posterior ischemic optic neuropathy in long cases with substantial blood loss [21]. These considerations support reserving awake techniques for procedures that can be performed with reliable somatosensory or no neuromonitoring and for cases in which the expected duration, blood loss, and positioning demands remain modest, while ensuring that every room has a rehearsed conversion algorithm and documentation of airway and neuromonitoring tradeoffs.

Additional caution is warranted in patients requiring repeated spine operations, where prior laminectomies, scars, and hardware can obscure landmarks and may modestly increase the risk of neuraxial technical difficulty or neurologic complications, even though neuraxial blockade can often still be performed successfully in experienced hands [22]. In contrast, emergent scenarios such as acute spinal cord compression, polytrauma, or hemodynamic instability will almost always remain the domain of general anesthesia, given the need for rapid imaging, expeditious decompression, and unrestricted neuromonitoring, in line with current consensus guidance for traumatic spinal cord injury [23]. Prospective comparative trials and pragmatic registries should therefore focus on clearly elective indications, prespecify neuromonitoring strategies and conversion criteria, and collect granular data on postoperative pain, opioid consumption, nausea, time to ambulation, and patient-reported satisfaction [24], so that awake pathways may be tailored to those patients and procedures most likely to benefit while respecting the genuine physiologic and logistical limits of the technique.

### 4.4. Limitations

This umbrella review synthesizes diverse studies, enhancing external relevance while introducing variability that must be recognized. This umbrella review was not prospectively registered in a public registry, which may constitute a limitation with respect to documenting our protocol a priori. The pooled gains in length of stay, operative time, and blood loss were substantial on average, but between-study heterogeneity was high and prediction intervals included values compatible with smaller or absent effects, indicating considerable variation across settings. Awake techniques also showed statistically significant pooled effects for postoperative nausea and vomiting and postoperative analgesic requirements, although these outcomes were graded with lower credibility and had prediction intervals that encompassed the null, consistent with context-dependent effects. Intraoperative hypotension did not differ significantly between pathways, and the high heterogeneity with wide prediction intervals suggests that blood pressure patterns vary by local management and case mix. Bias diagnostics showed small-study effects and excess significance for selected endpoints only, and credibility grading placed efficiency outcomes in an intermediate tier and most other associations in a lower tier. Additionally, there were differences in intrathecal and systemic opioid regimens across trials, including the use of neuraxial opioids in some spinal protocols, which may modify observed differences in postoperative pain, PONV, and analgesic requirements. Taken together, the direction of effects across multiple domains consistently favors awake pathways and supports their use as a promising strategy in selected patients, but the heterogeneous and often low-credibility evidence base indicates that larger, protocolized multicenter trials and prospective registries are needed to refine effect sizes, test generalizability, and define the procedural and institutional contexts in which awake lumbar surgery provides the most reliable benefits.

## 5. Conclusions

In summary, across comparative studies of lumbar procedures, awake anesthesia was associated with a shorter length of stay, shorter operative time, lower intraoperative blood loss, and reduced postoperative nausea, vomiting, and analgesic requirements. These advantages are biologically plausible given the avoidance of induction, deeper anesthetic planes, and airway manipulation, and they may translate into practical gains in perioperative efficiency and symptom control when pathways are mature and protocolized. At the same time, confidence intervals and prediction intervals were wide, intraoperative hypotension and bradycardia did not differ consistently between pathways, and overall complication rates and pain scores were similar, indicating that the benefits achieved vary with local case mix, team experience, and perioperative protocols. Taken together, the findings support the adoption of awake pathways in settings where efficiency gains and opioid and PONV reduction have high clinical value, especially for ambulatory decompressions and other straightforward cases, while pairing implementation with clear conversion criteria and routine auditing of patient-reported outcomes and time stamps. Future work should emphasize larger, prospective, protocolized comparisons, harmonized outcome definitions, and systematic evaluation of moderators such as procedure type, prior surgery, sedation strategy, neuromonitoring use, and enhanced recovery participation so that effect sizes are refined and the patients most likely to benefit are identified with greater precision.

## Figures and Tables

**Table 1:** Study characteristics across included meta-analyses.

References	Last Search	Data Sources	Publication Date Range of the Primary Studies	Primary Study Designs	Total Number of Primary Studies	Number of Pooled Patients	Number of Pooled Awake Patients	Number of General Anesthesia Patients	Reported Outcomes of Interest	Surgery Type/Region
Weber 2025 [1]	Not Reported	Cochrane Library, Ovid MEDLINE, Ovid Embase	1996–2024	Prospective and retrospective comparative studies	11	1350	688	662	Operative time, intraoperative blood loss, LOS, postoperative pain, postoperative complications, postoperative nausea and vomiting	Lumbar
Rajjoub 2024 [14]	14 April 2023	Ovid MEDLINE, Ovid Embase, Cochrane Central, and Scopus	1996–2023	RCTs, prospective and retrospective comparative studies	38	7820	5061	2759	Operative time, intraoperative blood loss, LOS, postoperative complications	Thoracic and lumbar
Jadczak 2023 [12]	March 2021	Embase, PubMed/Medline, Cochrane, and Google Scholar	2011–2020	RCTs, prospective and retrospective comparative studies	26	2113	1873	240	Postoperative complications, LOS, PROMS	Endoscopic
Shui 2023 [15]	31 May 2020	Embase, PubMed, Cochrane	1995–2014	RCTs	10	733	367	366	Intraoperative hemodynamics (hypertension, hypotension, tachycardia, bradycardia), analgesic requirements (PACU, 24h after surgery), postoperative nausea and vomiting, urinary retention, headache, LOS	Lumbar
Perez-Roman 2021 [2]	NR	PubMed and Cochrane	2003–2021	RCTs, prospective and retrospective comparative studies	14	3709	2219	1490	Operative time, anesthesia time, postoperative complications, postoperative pain, LOS	Lumbar
De Cassai 2020 [13]	6 July 2020	PubMed, Cochrane, and Google Scholar	1996–2020	RCTs	11	896	447	449	Postoperative pain, analgesic requirements, blood loss, operative time, intraoperative hemodynamics (hypotension and bradycardia), postoperative nausea and vomiting, urinary retention, LOS, PROM	Lumbar
Meng 2017 [11]	1 July 2016	PubMed, Embase, Cochrane	1996–2014	RCTs	8	625	313	312	Intraoperative hemodynamics (hypertension, hypotension, tachycardia, bradycardia), blood loss, operative time, analgesic requirements, postoperative nausea and vomiting, LOS	Lumbar

**Table 2:** AMSTAR2 tool assessment of included meta-analyses.

Meta-Analysis	Uses Elements of PICO	Explained Selection of the Study Designs	Comprehensive Literature Search	Study Selection in Duplicate	Excluded Study List Provided	Included Studies Described	Funding Sources Reported	Quality Assessed	Quality Used Appropriately	Satisfactory Discussion of Heterogeneity	Conflicts of Interest Reported	AMSTAR2
Weber 2025 [1]	X	X	X	X	X	X	X	X	X	X	X	11
Rajjoub 2024 [14]	X	X	X	X	X	X	X	X	X		X	10
Jadczak 2023 [12]	X	X	X	X	X	X		X	X			9
Shui 2023 [15]	X	X	X	X	X	X		X	X		X	9
Perez-Roman 2021 [2]	X	X	X	X	X	X		X	X		X	9
De Cassai 2020 [13]	X	X	X	X	X	X	X	X	X	X	X	11
Meng 2017 [11]	X	X	X	X	X	X	X	X	X	X		10
Total	7	7	7	7	7	7	4	7	7	3	5	9.86

Note. X = included study met the corresponding AMSTAR2 criterion.

**Table 3:** Outcomes across included meta-analyses.

Outcome	Meta-Analysis	Number of Primary Studies	Number of Pooled Patients	Number of Pooled Awake Patients	Number of Pooled General Anesthesia Patients	Effect Measure	Effect Size	95% CI	*p*-Value for Effect (* Computed)	*I*^2^ (%)	*p*-Value for Heterogeneity (* Computed)
Operative time	Weber 2025 [1]	8	1138	574	564	MD	−8.52	[−14.56, −2.49]	0.0056 *	93.49	<0.0001 *
	Rajjoub 2024 [14]	18	3101	1661	1440	MD	−19.17	[−29.68, −8.65]	0.00036 *	98	<0.01
	Perez-Roman 2021 [2]	5	1793	1281	512	MD	−14.04	[−21.30, −6.79]	0.0001	88	<0.00001
	De Cassai 2020 [13]	10	830	414	416	MD	−4.56	[−12.16, 4.04]	0.3	98	<0.01 *
	Meng 2017 [11]	6	503	252	251	SMD	0.41	[−1.73, 0.91]	0.54	98	<0.00001 *
Intraoperative blood loss	Weber 2025 [1]	6	695	347	348	MD	−27.59	[−61.85, 6.67]	0.114 *	99.77	<0.00001 *
	Rajjoub 2024 [14]	18	3101	1661	1440	MD	−19.17	[−29.68, −8.65]	0.00036 *	98	<0.01
	De Cassai 2020 [13]	6	554	286	268	MD	−53.88	[−98.13,−9.63]	0.02	97	<0.00001
	Meng 2017 [11]	5	434	216	218	SMD	1.56	[−3.12, 0.00]	0.05	98	<0.00001 *
Length of stay (LOS)	Weber 2025 [1]	3	343	171	172	MD	−1.6	[−3.95, 0.75]	0.182 *	99.89	<0.00001
	Rajjoub 2024 [14]	13	1915	807	1108	MD	−0.4	[−0.64, −0.17]	0.00085 *	81	<0.01
	Jadczak 2023 [12]	3	283	163	120	MD	−2.09	[−3.99, −0.19]	0.03	99.39	0.00
	Shui 2023 [15]	6	478	239	239	MD	−0.28	[−0.37, −0.18]	<0.00001	32	0.2
	Perez-Roman 2021 [2]	4	794	412	382	MD	−0.16	[−0.29 to −0.03]	0.02	0	0.58
	De Cassai 2020 [13]	7	578	289	289	MD	−0.31	[−0.41, −0.21]	<0.00001	54	0.04
	Meng 2017 [11]	3	258	131	131	SMD	−1.15	[−1.98, −0.31]	0.007	89	0.00011 *
Postoperative pain (VAS)	Weber 2025 [1]	2	172	86	86	MD	−0.22	[−1.35, 0.92]	0.705 *	88.26	0.0035 *
	Perez-Roman 2021 [2]	2	604	391	213	MD	−2.50	[−3.91, −1.09]	0.0005	97	0.00001
	De Cassai 2020 [13]	4	306	153	153	SMD	−0.33	[−0.69, 0.04]	0.08	60	0.06
Postoperative complications	Weber 2025 [1]	5	928	443	485	RR	0.86	[0.75, 0.99,]	0.033	70.72	0.0085
	Jadczak 2023 [12]	6	743	348	395	OR	0.97	[0.22, 4.41]	0.97	87.4	0.00
	Perez-Roman 2021 [2]	6	2334	1642	692	OR	0.18	[0.09, 0.35]	<0.00001	79	0.0002
Postoperative nausea and vomiting (PONV)	Weber 2025 [1]	5	845	428	417	RR	0.58	[0.51, 0.66]	<1 × 10^−15^ *	0	0.41 *
	Shui 2023 [15]	14	172	51	121	OR	0.34	[0.18, 0.66]	0.001	60	0.002
	De Cassai 2020 [13]	10	796	397	399	OR	2.69	[1.73, 4.20]	<0.0001	24	0.22
	Meng 2017 [11]	7	545	273	272	RR	0.29	[0.18, 0.46]	<0.00001	12	0.34
Analgesic requirements	Shui 2023 [15]	8	380	151	229	OR	0.32	[0.13, 0.77]	0.01	80	<0.0001
	De Cassai 2020 [13]	6	534	266	266	OR	11.52	[5.12, 25.93]	<0.00001	57	0.04
	Meng 2017 [11]	4	362	181	181	RR	0.32	[0.24, 0.43]	<0.00001	0	0.96
Intraoperative hypotension	Shui 2023 [15]	5	388	194	194	OR	1.11	[0.68, 1.81]	0.68	0	0.61
	De Cassai 2020 [13]	7	580	289	291	OR	0.51	[0.23, 1.11]	0.09	61	0.02
	Meng 2017 [11]	5	428	214	214	RR	1.48	[0.75, 2.93]	0.26	73	0
Intraoperative bradycardia	Shui 2023 [15]	5	422	211	211	OR	0.95	[0.55, 1.62]	0.84	0	0.42
	De Cassai 2020 [13]	6	520	259	261	OR	0.74	[0.30, 1.80]	0.51	55	0.05
	Meng 2017 [11]	5	428	214	214	RR	0.87	[0.57, 1.31]	0.5	19	0.29

Note. MD = Mead Difference. SMD = Standardized Mean Difference. RR = Relative Risk. OR = Odds Ratio. Note. * = computed p values (effect and heterogeneity) derived from reported effect sizes and 95% CIs; *I*^2^ values are taken from the source studies.

**Table 4:** Summary of umbrella pooled analysis across outcomes.

**a**												
**Summary Results**												
**Factor**		**Criteria**		**Class**	**n_Studies**		**Total_n**		**n_Cases**		**n_Controls**	
Length of stay		Ioannidis		III	22		3965		2090.0		1875.0	
Operative time		Ioannidis		III	38		7029		4108.0		2921.0	
Analgesic requirements		Ioannidis		IV	7		520		121.0		399.0	
Intraoperative blood loss		Ioannidis		IV	15		2721		1382.0		1339.0	
Postoperative nausea and vomiting		Ioannidis		IV	14		279		70.0		209.0	
Intraoperative bradycardia		Ioannidis		ns	7		150		68.0		82.0	
Intraoperative hypotension		Ioannidis		ns	10		336		174.0		162.0	
Postoperative pain (VAS)		Ioannidis		ns	8		672		287.0		385.0	
Surgical complications		Ioannidis		ns	9		188		77.0		111.0	
**b**												
**Factor**	**Measure**	**Value**	**Value_*CI***		**eG**	**eG_*CI***		**eOR**		**eOR_*CI***		***p*** **_Value**
Length of stay	G	−1.003	[−1.522, −0.484]		−1.003	[−1.522, −0.484]		0.162		[0.063, 0.415]		1.50 × 10^−4^
Operative time	G	−0.773	[−1.197, −0.349]		−0.773	[−1.197, −0.349]		0.246		[0.114, 0.531]		3.57 × 10^−4^
Analgesic requirements	OR	0.093	[0.037, 0.234]		−1.310	[−1.819, −0.801]		0.093		[0.037, 0.234]		4.58 × 10^−7^
Intraoperative blood loss	G	−1.708	[−3.081, −0.335]		−1.708	[−3.081, −0.335]		0.045		[0.004, 0.544]		1.47 × 10^−2^
Postoperative nausea and vomiting	OR	0.376	[0.228, 0.618]		−0.540	[−0.814, −0.265]		0.376		[0.228, 0.618]		1.16 × 10^−4^
Intraoperative bradycardia	OR	0.977	[0.512, 1.864]		−0.013	[−0.369, 0.343]		0.977		[0.512, 1.864]		9.43 × 10^−1^
Intraoperative hypotension	OR	1.008	[0.459, 2.214]		0.004	[−0.429, 0.438]		1.008		[0.459, 2.214]		9.84 × 10^−1^
Postoperative pain (VAS)	G	−2.189	[−5.338, 0.96]		−2.189	[−5.338, 0.96]		0.019		[0, 5.704]		1.73 × 10^−1^
Surgical complications	OR	0.828	[0.456, 1.501]		−0.104	[−0.432, 0.224]		0.828		[0.456, 1.501]		5.33 × 10^−1^

Note. value_CI = value of the effect size and 95% confidence interval. Note. eG = equivalent Hedges’ G//eOR = equivalent odds ratio.

## Data Availability

Data are available upon request.

## Supplementary Materials

The following supporting information can be downloaded at https://www.mdpi.com/article/10.3390/jcm14238335/s1, Table S1: PRISMA 2020 Checklist.
